# Predictive encoding of pure tones and FM-sweeps in the human auditory cortex

**DOI:** 10.1093/texcom/tgac047

**Published:** 2022-11-16

**Authors:** Jasmin Stein, Katharina von Kriegstein, Alejandro Tabas

**Affiliations:** Faculty of Psychology, Technical University Dresden, Dresden 01062, Germany; Chair of Cognitive and Clinical Neuroscience, Faculty of Psychology, Technical University Dresden, Bamberger Str. 7, Dresden 01187, Germany; Chair of Cognitive and Clinical Neuroscience, Faculty of Psychology, Technical University Dresden, Bamberger Str. 7, Dresden 01187, Germany

**Keywords:** predictive coding, auditory cortex, FM-sweeps, fMRI

## Abstract

Expectations substantially influence perception, but the neural mechanisms underlying this influence are not fully understood. A prominent view is that sensory neurons encode prediction error with respect to expectations on upcoming sensory input. Although the encoding of prediction error has been previously demonstrated in the human auditory cortex (AC), previous studies often induced expectations using stimulus repetition, potentially confounding prediction error with neural habituation. These studies also measured AC as a single population, failing to consider possible predictive specializations of different AC fields. Moreover, the few studies that considered prediction error to stimuli other than pure tones yielded conflicting results. Here, we used functional magnetic resonance imaging (fMRI) to systematically investigate prediction error to subjective expectations in auditory cortical fields Te1.0, Te1.1, Te1.2, and Te3, and two types of stimuli: pure tones and frequency modulated (FM) sweeps. Our results show that prediction error is elicited with respect to the participants’ expectations independently of stimulus repetition and similarly expressed across auditory fields. Moreover, despite the radically different strategies underlying the decoding of pure tones and FM-sweeps, both stimulus modalities were encoded as prediction error in most fields of AC. Altogether, our results provide unequivocal evidence that predictive coding is the general encoding mechanism in AC.

## Introduction

Subjective expectations influence our perception of the world (De Lange et al. 2018). They facilitate perceiving noisy (Chalk et al. 2010; Leonard et al. 2016) or ambiguous (Chambers et al. 2017; Sterzer et al. 2008) sensory input, and bias perception when we hold strong expectations about the sensory world (Lange 2009). Understanding the mechanisms integrating our expectations with the sensory input is an essential prerequisite to understand perception. The predictive coding framework is a theory of sensory processing aiming to describe these mechanisms. Its main tenet is that sensory neurons encode prediction error with respect to an internal generative model of the sensory world (Friston 2003; Mumford 1992; Rao and Ballard 1999; Spratling 2017).

Neurons in the rodent auditory cortex (AC) encode pure tones as prediction error (Nieto-Diego and Malmierca 2016; Parras et al. 2017; Pérez-González et al. 2021; Rubin et al. 2016). Prediction error is typically elicited using oddball paradigms, where predictable repetitions of a standard sound are rarely interrupted by a deviant. Individual neurons in the AC show reduced responses to repeated standards and recovered responses to deviants, a phenomenon that is called stimulus-specific adaptation (SSA; Ulanovsky et al. 2003). SSA is typically interpreted as prediction error (Malmierca 2015). However, it can also been explained in terms of neural habituation (Eytan et al. 2003; Mill et al. 2011; Wang et al. 2014; see Tabas et al. 2021a for review, and Carbajal and Malmierca 2018 for a different perspective). One way to disassociate whether SSA reflects habituation or prediction error is to manipulate participants’ subjective stimulus expectations on the sensory input orthogonally to stimulus repetition (Tabas et al. 2021a; Tabas et al. 2020).

Using abstract rules to induce subjective expectations, we have recently shown that SSA in neural populations of the subcortical auditory pathway reflects the encoding of prediction error (Tabas et al. 2021b; Tabas et al. 2020). Other studies have demonstrated similar effects in cortical areas measuring the mismatch negativity (MNN), a component of the event-related potentials assumed to encode prediction error (Cornella et al. 2012; Costa-Faidella et al. 2011; Dürschmid et al. 2016; Lecaignard et al. 2015; Phillips et al. 2016; Todorovic and de Lange 2012; Todorovic et al. 2011). However, since the MMN is at least partially generated in the frontal cortex (Deouell 2007; Shalgi and Deouell 2007), it is challenging to measure unambiguously what portion of the event-related potential stems from AC. Two studies using functional magnetic resonance imaging (fMRI) specifically investigated the encoding of prediction error in AC (Berlot et al. 2018; Cacciaglia et al. 2019). However, stimulus repetition and expectation were confounded in these studies. Thus, although the extended literature on prediction error in human AC seems to indicate that cortical SSA is also attributable to prediction error, this has not yet been formally tested: it is unclear whether prediction error is encoded by the same neural populations that exhibit SSA.

Additionally, previous studies did not consider in detail whether SSA or prediction error is similarly represented in distinct fields of the AC. The auditory system consists of a primary (lemniscal) and secondary (nonlemniscal) subdivision (Lee and Sherman 2011). Primary areas show narrow tuning curves; secondary areas are tuned more widely and support multisensory integration (Hu 2003). In rodents, SSA is stronger in secondary subdivisions of AC (Nieto-Diego and Malmierca 2016; Parras et al. 2017).

Furthermore, most previous research on the encoding of prediction error in AC has focused on static pure tones. However, natural auditory scenes often involve dynamic acoustic components; for instance, frequency modulation (FM) is a basic element of animal (Suga 2012) and human (Divenyi 2009) vocalization. In human speech, FM-sweeps constitute formant transitions, the main components of consonants preceding a vowel (Liberman et al. 1967), which are critical for phoneme identification.

Although we have previously investigated prediction error to FM in subcortical areas (Tabas et al. 2021b), whether FM is encoded as prediction error in the human AC is still unclear. Previous studies considered prediction error to FM in the human cerebral cortex and yielded mixed results: Some reported an MMN to deviating FM-stimuli, suggesting that FM is also encoded as prediction error (Cornella et al. 2013; Hsieh and Yeh 2021; Kung et al. 2020); others reported enhanced neural responses to repeated FM-stimuli, concluding that different predictive coding strategies underlie the encoding of pure tones and FM (Altmann et al. 2011; Heinemann et al. 2011; Heinemann et al. 2010; Okamoto and Kakigi 2017). This different encoding strategy might arise from the fact that, while pure tones are first encoded in the basilar membrane (Malmierca and Hackett 2010), FM selectivity is present only in the auditory midbrain, thalamus, and cortex (Altmann and Gaese 2014; Geis and Borst 2013; Hall et al. 2000; Hart et al. 2003; Issa et al. 2017; Lui and Mendelson 2003; Paltoglou et al. 2011). Studying whether FM is encoded as prediction error specifically in AC might shed light on these divergences.

Here, we systematically studied the encoding of prediction error to pure tones and FM-sweeps across fields of the human AC with high-resolution fMRI. We used a variation of the auditory oddball paradigm (Tabas et al. 2020) designed to manipulate participants’ expectations independently of local stimulus statistics. Our aim was to elucidate whether predictive coding is the general encoding mechanism in the human AC.

## Materials and methods

The studies were approved by the Ethics committee of the Medical Faculty of the University of Leipzig, Germany (pure tone experiment) and the Ethics committee of the Technische Universität Dresden, Germany (FM-sweep experiment). All participants provided written informed consent and received monetary compensation for their participation.

### Data acquisition and experimental design

We analyzed fMRI data from two experiments designed to study prediction error encoding of pure tones (Tabas et al. 2020) and FM-sweeps (Tabas et al. 2021b). Both experiments used the same variation of the auditory oddball paradigm, one with pure ones, one with FM-sweeps. They were both acquired at different scanning sites and using different participant cohorts.

#### Participants

All participants were neurotypical normal-hearing German native speakers (see Tabas et al. 2020 and Tabas et al. 2021b for further details on inclusion criteria). Nineteen participants (12 female) between the ages of 24 and 34 (average 26.6) participated in the pure tone study; 18 participants (12 female) between the age of 19 and 31 (average 24.6) participated in the FM-sweep study.

#### Stimuli

**Fig. 1:**
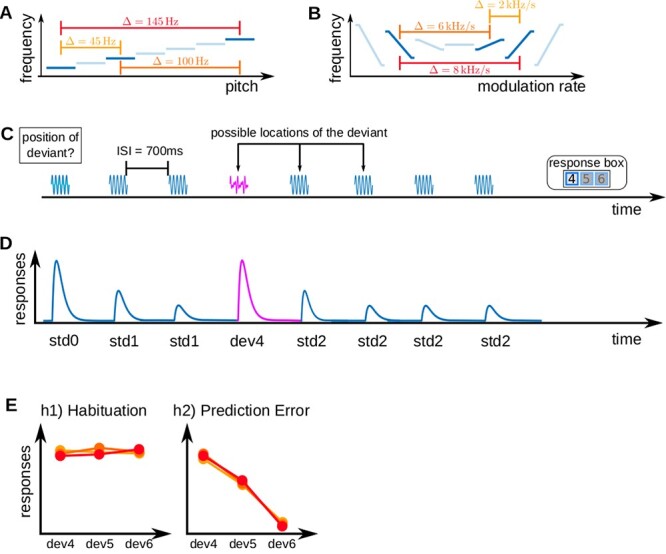
Experimental design and hypotheses. (A) Pure tone stimuli; three pure tones with different frequencies were used to build six standard-deviant combinations with three absolute frequency differences $\Delta = |f_{std} - f_{dev}|$. (B) FM-sweep stimuli; FM-sweeps were used to build standard-deviant combinations, characterized by the absolute difference between the frequency spans of standard and deviant sweeps ($\Delta = |\Delta f_{dev} - \Delta f_{std}|$). (C) An example of a trial sequence consisting of seven repetitions of a standard and one deviant stimulus. (D) Expected neural response to the exemplary trial shown in A (note the recovered response to the deviant as the main characteristic of SSA). (E) Two possible anticipated outcomes of the experiment; in h1 or habituation, it was assumed that high-level subjective expectations do not affect deviant responses; in h2 or prediction error, we expected sounds to be scaled by stimulus predictability and thus represent prediction error responses with respect to subjective expectations.

In the pure tone experiment, there were three pure tone stimuli of 50 ms duration (including 5 ms onset/offset ramps) and frequencies of 1455, 1500, and 1600 Hz. The tones were combined into six pairings of standard and deviant tones. Each of the resulting oddball sequences was consequently characterized by one of three possible absolute frequency differences characterizing the distance between standards and deviants ($\Delta = |f_{std} - f_{dev}|$ either 145, 100, or 45 Hz). See Fig. 1A for a visualization of sound stimuli and sequences.

In the FM-sweep experiment, there were three sinusoidal FM-sweeps with a duration of 50 ms (5 ms onset/offset ramps) and starting frequencies of 1000, 1070, or 1280 Hz. The FM-sweeps ended at either 1080, 1170, or 1200 Hz, respectively. The tones were also combined into six pairings of standard and deviant tones. An FM-sweep could deviate from the standard in its FM direction, FM rate, or both. Since all sweeps had the same duration, the defining property of the FM-sweeps was their frequency span $\Delta f$—the difference between starting and ending frequencies. Each of the sequences was consequently characterized by one of three possible absolute frequency span differences characterizing the distance between standards and deviants ($\Delta = |\Delta f_{dev} - \Delta f_{std}|$). See Fig. 1B for an exemplary illustration of the FM-sweep stimuli and sequences (Tabas et al. 2021b).

#### Experimental design

The design was a variation of the oddball paradigm where abstract rules were used to manipulate participants’ expectations on the upcoming stimuli independent of the local statistical regularity of the presented sound sequences. Specifically, participants listened to sequences of eight sounds: seven repeated standards and one deviant that could occur in positions four, five, or six. The stimuli were separated by a 700 ms interstimulus interval.

Participants were told explicitly that all sequences contained a deviant and that the deviant could occur in positions four, five, or six. Participants were instructed to report the position of the deviant via button press as fast and as accurately as possible. Deviants were equally likely to be placed in each of the three positions. Thus, the probability of a deviant in position four after hearing three standards was 1/3. However, if the deviant was not in position four, since deviants occur once in each sequence, the probability of hearing a deviant in position five after hearing four standards was 1/2. If the deviant was neither in position four nor five, the probability of a deviant in position six after hearing five standards was 1 (Tabas et al. 2021b; Tabas et al. 2020).

The intertrial interval was calculated so that the separation between deviants followed a Gaussian distribution $\mathcal{N}(\mu = 5\, s, \sigma = 1\, s)$ to maximize the statistical power of the estimation of the responses to the deviants (Friston et al. 1999). The ITI was further truncated so that the minimum separation between trials was 1500 ms (i.e. the duration of two tones and two ITIs) to guarantee that participants could tell apart any two consecutive trials. This resulted in ITIs between 1500 and 11000 ms. All sound combinations were used the same number of times with all deviant locations across runs. Using three standard-deviant combinations reduced the effect of long-term adaptation effects across trials and rendered the task more engaging and less repetitive for the participants. The specific ordering of the trials was pseudorandomized independently for each participant to prevent potential effects of presentation ordering in the group results.

The pure tone experiment comprised four runs that were completed by all participants. The FM-sweep data were collected in three sessions with three runs each; most participants completed nine runs, one participant only completed eight due to technical reasons. We acquired a higher number of runs for the FM-sweep experiment to partially compensate for the lower SNR of the data: while the pure-tone experiment was performed using 7-Tesla fMRI at a tSNR of about 50, the FM-sweep experiment was performed using 3-Tesla fMRI at a tSNR of about 30. Fully compensating for the tSNR drop would have required to measure a 2.7-fold data; we measured as many runs as we could within our limited resources.

In both experiments, a run contained 6 blocks of 10 trials. Deviant positions were pseudo-randomized so that they all occurred 20 times in each run. The runs lasted for around 10 min and were separated by a 1 min break. Practice trials were presented at the beginning of the first run to ensure task understanding. Interspersed null events were used to optimize the fit of the GLMs (Friston et al. 1999). Further details can be found in Tabas et al. 2020 (pure tones) and Tabas et al. 2021b (FM-sweeps).

#### FMRI data acquisition

FMRI data were collected using EPI sequences and partial FoVs. Magnetic field strength and image resolution differed between data sets. Data from the pure tone experiment were collected using a Siemens Magnetom 7-Tesla scanner (Siemens Healthineers, Erlangen, Germany) with an 8-channel head coil and a voxel size of 1.5 mm isotropic; data from the FM-sweep experiment were collected using a Siemens Trio 3-Tesla scanner (Siemens Healthineers, Erlangen, Germany) with a 32-channel head coil and a voxel size of 1.75 mm isotropic. Interleaved slice acquisition was used in both data sets.

Pure tone data were collected using the following scanning parameters: $TR = 1600$ ms, $TE = 19$ ms, flip angle $65^{\circ }$, GRAPPA with acceleration factor 2, 33% phase oversampling, matrix size $88\times 88$, phase partial Fourier 6/8, $FoV = 132$ mm$\times $132 mm (30 slices). We also acquired three whole-head volumes with 80 slices to aid coregistration. FM-sweep data were collected using the following scanning parameters: $TR = 1900$ ms, $TE = 42.2$ ms, flip angle $66^{\circ }$, matrix size $88\times 88$, $FoV = 154$ mm$\times $154 mm (24 slices). We also acquired one whole head volumes with 84 slices to aid the coregistration process.

Structural images for the pure tone experiment were measured using an MP2RAGE T1 protocol (700 mm isotropic resolution, $TE = 2.45$ ms, TR = 5000$\,ms, TI1 = 900\, ms$, $TI2 = 2750$ ms, flip angle $1 = 5^{\circ }$, flip angle $2 = 3^{\circ }$, $FoV = 224$ mm$\times $224 mm, GRAPPA acceleration factor 2). Structural images for the FM-sweep data were measured using an MPRAGE T1 protocol (1 mm isotropic resolution, $TE = 1.95$ ms, $TR = 1000$ ms, flip angle $1 = 8^{\circ }$, $FoV = 256$ mm$\times $256 mm).

Physiological data (heart rate and respiration in the pure tone experiment, heart rate in the FM-sweep experiment) were collected and processed for use as regressors of no-interest during model estimation for both stimulus types.

### Data preprocessing

#### Anatomical data

Data preprocessing and analysis was implemented using Nipype 1.1.2 (Gorgolewski et al. 2011) and included functions from: the FMRIB Software Library, version 5 (FSL, Jenkinson et al. 2012); Freesurfer, version 7 (Fischl et al. 2002); the Advanced Normalization Tools, version 2.2.0 (ANTS, Avants et al. 2011); and the Statistical Parametric Mapping toolbox (SPM, Penny et al. 2011), version 12.

All anatomical data were resampled to a resolution of 1 mm isotropic. We computed the boundaries between gray and white matter using Freesurfer’s *recon-all*. These boundaries were later used for coregistration of the functional data to the participants’ structural images. In the case of the pure tone experiment, we first computed a brain mask excluding voxels containing air, cerebrospinal fluid, scalp, and skull. This was necessary because MP2RAGE (but not MPRAGE) yields noisy signals outside the brain that interfere with the automatic processes of *recon-all*. The mask was calculated using Freesurfer’s *BET* and SPM’s *Segment* and was applied using *FSLmath*. Then, Freesurfer’s *recon-all* was used to obtain gray and white matter boundaries, and ANTs was used to calculate the coregistration matrix between the anatomical data and the MNI152 symmetric template.

#### FMRI data

Functional data were slice time corrected using SPM’s *SliceTiming* using the first slice as reference volume. We used SPM’s *FieldMap Toolbox* to calculate distortions due to magnetic field heterogeneities. Then, motion and distortion correction was performed on the functional data separately for each session (SPM *Realign and Unwarp*). Nipype module’s *rapidart* was used to detect artifacts from the realigned functional data to serve as regressors of no-interest in our design matrix during GLM estimation. The resulting functional data were smoothed (SPM *Smooth*) using a 2 mm FWHM Gaussian kernel.

In the case of the pure tone data, the derivatives (i.e. log-evidences and beta maps) were registered to the anatomical space after fitting (see *GLM Estimation* and *Bayesian Model Comparison*). For FM-sweeps, the realigned functional data were registered to the anatomical space using Freesurfer’s *ApplyVolTransform* before model estimation to ensure all data were available in the same space during model fitting.

The transformation matrix between functional and structural data was computed using Freesurfer’s *BBRegister* using the white and gray matter boundaries computed as described above and the whole-brain EPI as an intermediate stage.

### Anatomical regions of interest

Anatomical regions of interest (ROIs) were taken from Morosan et al. (Morosan et al. 2001). The ROIs of interest were the bilateral auditory cortical fields Te1.0, Te1.1, Te1.2, and Te3 (Morosan et al. 2001). Areas Te1.0, Te1.1, and Te1.2 are located on Heschl’s gyrus (Te1.1 most medial, Te1.2 most lateral). Te1.0 and Te1.1 have been proposed to correspond mostly to BA 41. Te1.0 is generally considered representing the primary auditory cortex in humans (Moerel et al. 2014). Te1.2 overlaps with BA 42 (Moerel et al. 2014). Comparing human and primate auditory fields, it was assumed that Te1.0 corresponds to the auditory core and Te1.1 and Te1.2 represent medial junction and lateral belt (Moerel et al. 2014). Thus, Te1.1 is often considered an intermediate processing stage between primary and secondary auditory areas (Hackett et al. 2001). However, the correspondence of human and primate auditory fields is still unclear, e.g., Besle et al. 2019; Gulban et al. 2020; Moerel et al. 2014. Te3 is a secondary auditory association area lying on the lateral surface of the superior temporal gyrus as part of BA 22 and might correspond to parabelt areas in primates (Moerel et al. 2014).

### Data analysis

#### GLM estimation

First level analyses were performed using SPM’s *EstimateModel*. Statistical analysis at the participant- and group-level was conducted in MATLAB (The MathWorks Inc., Version 2020b) using custom code.

We estimated one GLM per participant. The model included six task regressors: $std0$ (the first standard in a sequence), $std1$ (standards before the deviant), $std2$ (standards after the deviant), $dev4$, $dev5$, and $dev6$ (deviants in positions four, five, and six). The first standard was modeled as a separate regressor to test for adaptation by comparing the estimates corresponding to $std0$ and $std1$/$std2$. Modelling $std1$ and $std2$ separately was necessary because we expected a slight recovery of neural responses to standards after the deviant (see Fig. 1D); without an informed hypothesis on how strong this recovery would be, we chose to set it as a free parameter of the model. In addition, we added physiological data, artifact regressors, and realignment parameters to the design matrix as regressors of no interest.

The regressors corresponding to $std1$ and $std2$ were linearly parametrically modulated according to the position of each sound within the sequence of each trial: values corresponding to each $std1$ sound were assigned amplitudes increasing from one to the total number of $std1$, and $std2$ sounds were assigned increasing amplitudes from one to the total number of $std2$ in each trial. For example, in a sequence with a deviant in position four, the two $std1$ sounds were assigned the amplitudes amp$_{1} = [1, 2]$ and the four $std2$ sounds were assigned the amplitudes amp$_{2} = [1, 2, 3, 4]$. The parametric modulation indexes the standards, and it is meant to allow SPM a greater flexibility fitting the regressors under the assumption that the responses might vary linearly with successive repetition of the standard. We used this technique to circumvent the problem that responses to individual standards cannot be accurately fitted due to their temporal proximity. Since SPM has no biases for negative or positive slopes or finite or zeroed intercepts, using the indices was the most parsimonious way to declare the linear fit. All amplitudes were z-standardized before model fitting.

Model estimations for the pure tone data were done using the smoothed data in the native space of the functional data of the individual participants. For FM-sweeps, we estimated the models in the space of the participants’ anatomical scans. After model estimation, the spatial transformations calculated before were applied to the resulting statistical maps. The statistical maps of the pure tone data were first registered to the participants’ anatomical scans using Freesurfer’s *ApplyVolTransform* and subsequently to the MNI152 symmetric template using ANTs’ *ApplyTransforms*. For FM-sweeps, statistical maps were registered directly to MNI space.

The resulting beta estimates were $z$-standardized according to participant, experimental run, and ROI before the second level analyses to reduce variance specific to participants, runs, and ROIs.

#### Identifying voxels showing SSA

To localize voxels showing SSA, we first identified voxels within the anatomical ROIs showing adaptation (reduced responses to repeated standards) and deviant detection (stronger response to deviants compared with standards) as defined by commonly used indices from animal literature (Parras et al. 2017): the repetition suppression and neuronal mismatch indices, respectively. Adapting voxels were identified using the repetition suppression index, in our study defined by the contrast $std0> 0.5\, std1 + 0.5\, std2$. This assumes that the responses to the first standard, $std0$, were unaffected by habituation. This was a possible in our design because, unlike in the animal literature where oddball sequences last for several minutes, we used numerous short trials that allowed us to accurately estimate the responses to the first, unadapted standard.

Deviant detecting voxels were identified using the neuronal mismatch index, in our study defined by the contrast $dev4> 0.5\, std1 + 0.5\, std2$ (cf., with the animal literature index $dev> std$). Since we had two estimations of the adapted standards, $std1$ and $std2$, we calculated their average. We only included $dev4$ (excluding $dev5$ and $dev6$) because the effects of abstract expectations, absent in animal studies, were expected to be the lowest in $dev4$. We tested both contrasts using right-tailed rank-sum tests. Before conducting the rank-sum tests, we averaged single-voxel beta estimates for each experimental condition across all experimental runs.

We defined SSA regions for each stimulus type as the set of voxels showing significant adaptation and deviant detection; namely, voxels that adaptated to frequently occurring sounds while showing preserved responses to deviants (Ulanovsky et al. 2003). We computed voxel-wise $P$-values for SSA as the maximum of the uncorrected $P$-values for adaptation and deviant detection in each voxel; $P_{SSA} = max(P_{adaptation}, P_{deviant\ detection})$. All voxels’ $P$-values were subsequently controlled for the false discovery rate (FDR) using the Benjamini–Hochberg method (Benjamini and Hochberg 1995) and thresholded at $\alpha = 0.05$. Peak-level $P$-values were corrected for the FWE rate: we corrected for the number of voxels per ROI using Bonferroni-correction and for the total number of comparisons using the Holm–Bonferroni method (Holm 1979).

#### Quantifying SSA magnitude

To quantify SSA magnitude in each voxel of the anatomical ROIs, we computed the standardized voxel-wise index of SSA (SSAi) (Ulanovsky et al. 2003) following the procedure described in previous research (Tabas et al. 2021b; Tabas et al. 2020). We normalized the beta estimates for $dev4$, $std1$, and $std2$ to a range from zero to one, averaged these values in each voxel across participants and runs, and computed the index of SSA as $\textrm{SSAi} = (dev4 - 0.5\, std1 - 0.5\, std2) / (dev4 + 0.5\, std1 + 0.5\, std2)$.

#### Classical analysis

For both stimulus types, we conducted a classical statistical analysis to test for differences between responses to deviants in positions four, five, and six. To specifically investigate mechanisms driving SSA, we restricted this analysis to SSA clusters with significant ($P < 0.05$, FWE-corrected) peak-level $P$-values—the SSA ROIs.

We tested the pairwise differences between responses to deviants in different positions ($dev4> dev5$, $dev4> dev6$, and $dev5> dev6$) in each SSA ROI using one-sided Wilcoxon sign rank tests at the group-level. Before testing the contrasts, we averaged data corresponding to each experimental condition across runs and voxels within each participant and SSA ROI. In line with the idea of prediction error encoding, we expected deviant responses to be stronger when deviants are less expected.

We also measured the effect size of adaptation and deviant detection by testing the contrasts $std0> std2$ and $dev4> std2$. Note that these contrasts are not independent of the contrasts used for SSA voxel selection. However, we included them here to be able to quantify the size of both effects. Additionally, we included the comparison of $dev6$ and $std2$ using two-tailed Wilcoxon sign rank tests. We included this analysis to test whether responses to fully predictable deviants were comparable to standard responses. In line with predictive coding, we expected no statistically significant difference between responses to $dev6$ and $std2$. That is because both types of sound are fully predictable given our experimental design. All $P$-values were corrected for multiple comparisons using the Holm–Bonferroni method (Holm 1979).

#### Correlational analysis and linear mixed-effects model

To investigate the hypothesized negative relationship between deviant predictability and deviant responses further, we estimated a linear mixed-effects model (LMM) in each SSA ROI for each data set at the group-level. For pure tones, the model included deviant predictability as a fixed effect and random intercepts and slopes for experimental runs and participants

$$ \begin{align*} & \textrm{beta} \sim 1 + \textrm{predictability} + (1 + \textrm{predictability}|\textrm{run}) +\\& (1 + \textrm{predictability}|\textrm{participant}). \end{align*} $$

For FM-sweeps, we used the same model but added experimental session as an additional random effect. All $P$-values were Bonferroni-corrected for the total number of SSA ROIs. To test if the group-level results were replicated at the participant-level, we computed Spearman’s rank correlation between deviant predictability (1/3 for $dev4$, 1/2 for $dev5$, and 1 for $dev6$) and standardized beta estimates for $dev4$, $dev5$, and $dev6$ in each participant. Before computing the correlation coefficient $\rho $, beta estimates for the different deviant conditions in each voxel were averaged across experimental runs.

#### Bayesian model comparison

We constructed two models, each representing one potential encoding mechanism driving SSA (see Fig. 1E). The models were defined using parametric amplitude modulation vectors that specified the predicted responses to all tones in each trial.

**h1) Habituation:** SSA is based on stimulus repetition. Responses to standards undergo habituation over time and recover slightly after the deviant. Deviant responses are fully recovered and do not differ between deviants of differential predictability.

The h1 model (see Fig. 1E, left) was specified by assigning the amplitude 1 to *std0* and the deviant of a sequence. Standards before the deviant were assigned the amplitudes $1/n$ and standards after the deviant were assigned the amplitudes $1/(n - 1)$, where $n$ is the position of the standard within the sequence (see Table 1 for the exact amplitudes). Amplitudes were chosen based on a large body of literature demonstrating that adaptation in the AC is best described as exponential decay (e.g., Bäuerle et al. 2011; Ulanovsky et al. 2004).

**Table 1:** Amplitudes of the models used for Bayesian model comparison. h1 (habituation): asymptotically decreasing responses to repeating standards, full responses to deviants, and a slight recovery after the deviant; h2 (prediction error): neural responses scaled by sound predictability, amplitude defined as the probability $P$ of finding the heard sound in each position of the sequence.

**h1**	deviant position	1	2	3	4	5	6	7	8
	4	1	1/2	1/3	1	1/3	1/4	1/5	1/6
a$_{0}$	5	1	1/2	1/3	1/4	1	1/4	1/5	1/6
	6	1	1/2	1/3	1/4	1/5	1	1/5	1/6
**h2**	**deviant position**	**1**	**2**	**3**	**4**	**5**	**6**	**7**	**8**
	4	1/2	1	1	1/3	1	1	1	1
a$_{0}$	5	1/2	1	1	2/3	1/2	1	1	1
	6	1/2	1	1	2/3	1/2	1	1	1

**h2) Prediction error:** Neural responses to sounds are scaled by stimulus predictability and thus represent prediction error responses, which are stronger when stimuli are less expected.

The h2 model (see Fig. 1E, right) used an amplitude of 0.5 for $std0$ and an amplitude of $P$ ($P$ = probability of stimulus occurrence) for the rest of the tones. For example, a sequence with a deviant in position five was assigned the amplitude vector amp$_{0} = [1/2, 1, 1, 2/3, 1/2, 1, 1, 1]$. Thus, $std4$ was assigned a value of $2/3$ since a standard in position four is expected with a probability of $2/3$, and $dev5$ was assigned a value of $1/2$ because deviants in position five are expected with a probability of $1/2$ after hearing four standards. This definition corresponds to the established definition of precision-weighted prediction error (Friston 2003). See Table 1 for all amplitudes of h2. Since model estimation using parametric modulation is symmetric with respect to linear transformations of the amplitudes, the above-described amplitudes are equivalent to assuming a decreasing response with increasing sound predictability. We disregarded the trial information on the deviant-standard combination because the predictability of the deviant was the regressor that best separated the habituation and prediction error hypotheses. Adding additional regressors might have helped fine-tuning the definitions of the models, but we preferred to tailor our analysis to solve our specific research question.

For each subject, we computed the log-evidence of the two models in each voxel of all anatomical ROIs using SPM’s Bayesian Estimation functions in Nipype. Before model fitting, the amplitudes of all models were $z$-standardized according to experimental runs.

For pure tones, all models were estimated using the smoothed functional data in their native space. The log-evidence maps were registered to the individual T1 scans and then to the MNI152 symmetric template using Freesurfer’s *ApplyVolTransform* and ANTs’ *ApplyTransforms*, respectively. We combined the log-evidence-maps of all participants and calculated posterior probability maps for each model using previously written custom code (Tabas et al. 2020) following the methodology described elsewhere (Rosa et al. 2010; Stephan et al. 2009).

For FM-sweeps, all models were estimated using the smoothed functional data across sessions in the space of the participants’ anatomical scans. The resulting log-evidence-map of each participant was registered to the MNI152 symmetric template using ANTs’ *ApplyTransforms* and posterior probability maps were calculated as described before.

To compare the posterior likelihood of the two models (h1 and h2), we computed the Bayes factor $K$ for each stimulus type in each voxel of each anatomical ROI. We plotted Bayes factor maps (i.e., $K_{h2/h1}$) as an indicator of the strength of evidence in favor of h2, our model of interest.

## Results

### Topography of SSA to pure tones and FM-sweeps in AC

**Fig. 2:**
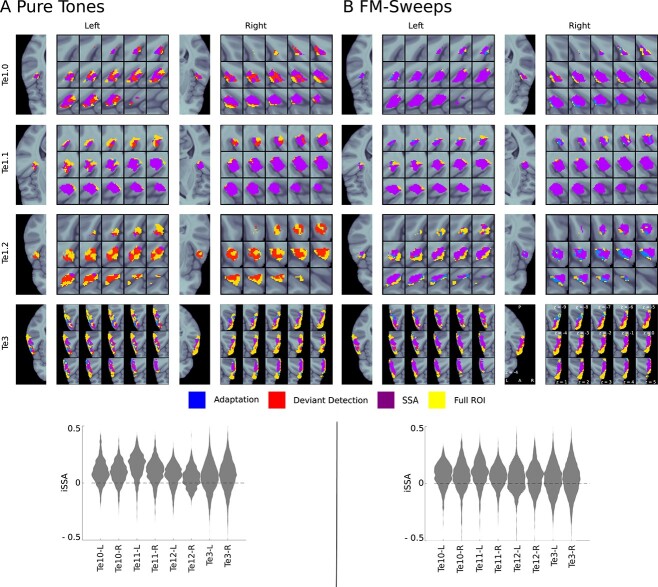
Adaptation, deviant detection, and SSA regions. Upper panels: voxels exhibiting significant ($P < 0.05$, FDR-corrected) adaptation (blue indicates adaptation alone, purple indicates SSA which entails adaptation), deviant detection (red indicates deviant detection alone, purple indicates SSA which entails deviant detection), and SSA (purple) are shown for each anatomical ROI (yellow). Lower panels: Distributions of SSA magnitude in all anatomical ROIs; we found very similar distributions in all ROIs and for both stimulus types. (A) Pure tones; (B) FM-sweeps.

There was a significant SSA to pure tones in bilateral Te1.0, bilateral Te1.1, and right Te3 ($P < 0.008$, FWE-corrected, see Table 2). Significant SSA clusters were present in all anatomical ROIs for FM-sweeps ($P < 0.03$, FWE-corrected, see Table 2). SSA clusters formed coherent fields for both stimulus types, indicating a systematic spatial encoding pattern (see Fig. 2 for a detailed map of adapting, deviant detecting, and SSA voxels).

**Table 2:** Cluster sizes, MNI peak coordinates (mm), and peak-level $P$-values for adaptation, deviant detection, and SSA to pure tones and FM-sweeps. Voxel-wise $P$-values were FDR-corrected in each ROI and thresholded at $\alpha = 0.05$. Peak-level $P$-values were corrected for the number of voxels per ROI and the total number of comparisons using Bonferroni– and Holm–Bonferroni-correction, respectively. Maps showing voxels in which the respective contrasts were significant are provided in Fig. 2.

		Pure Tones			FM-Sweeps		
Contrast	ROI	Size	Coordinates	Peak $P$	Size	Coordinates	Peak $P$
Adaptation	Te1.0 L	539	$[-48,-25, 9]$	0.007	950	$[-42,-20, 5]$	0.008
	Te1.0 R	759	$[ 54,-16, 7]$	0.003	1085	$[ 54,-15, 4]$	0.009
	Te1.1 L	920	$[-37,-33, 14]$	0.003	1145	$[ 39,-23, 5]$	0.008
	Te1.1 R	1374	$[ 35,-29, 18]$	0.003	1528	$[-50,-11, -1]$	0.009
	Te1.2 L	118	$[-48,-13, 2]$	0.008	715	$[ 51, -4, -4]$	0.02
	Te1.2 R	119	$[ 57, -3, -6]$	0.07	730	$[ 51, -4, -4]$	0.01
	Te3 L	4002	$[-65,-19, 9]$	0.02	4747	$[-58,-14, -4]$	0.02
	Te3 R	3688	$[ 63,-22, 0]$	0.008	4200	$[ 64,-28, 4]$	0.02
Deviant Detection	Te1.0 L	788	$[-48,-25, 10]$	0.003	908	$[-48,-22, 7]$	0.008
	Te1.0 R	1042	$[ 54,-16, 7]$	0.002	1015	$[ 51,-18, 5]$	0.008
	Te1.1 L	1000	$[-38,-32, 13]$	0.002	1114	$[-38,-24, 8]$	0.008
	Te1.1 R	1532	$[ 38,-22, 6]$	0.003	1424	$[ 48,-23, 7]$	0.008
	Te1.2 L	619	$[-53, 2, -3]$	0.003	631	$[-51,-11, -1]$	0.008
	Te1.2 R	462	$[ 54, -1, -3]$	0.02	547	$[ 57, -4, -4]$	0.008
	Te3 L	3128	$[-62,-21, 9]$	0.01	3954	$[-66,-19, 1]$	0.03
	Te3 R	3239	$[ 65,-22, 1]$	0.007	3901	$[ 63,-28, 4]$	0.02
SSA	Te1.0 L	499	$[-48,-25, 9]$	0.006	906	$[-48,-22, 8]$	0.008
	Te1.0 R	748	$[ 54,-16, 7]$	0.002	995	$[ 51,-18, 6]$	0.007
	Te1.1 L	88	$[-37,-33, 14]$	0.003	1092	$[-38,-24, 8]$	0.007
	Te1.1 R	1372	$[ 35,-30, 18]$	0.005	1413	$[ 41,-28, 14]$	0.007
	Te1.2 L	82	$[-48,-13, 3]$	0.06	570	$[-51, -9, -1]$	0.02
	Te1.2 R	0	-	-	531	$[ 51, -4, -4]$	0.01
	Te3 L	2435	$[-63,-20, 10]$	0.05	3586	$[-63,-15, -3]$	0.02
	Te3 R	2439	$[ 65,-22, 1]$	0.007	3351	$[ 66,-20, 6]$	0.02

**Fig. 3:**
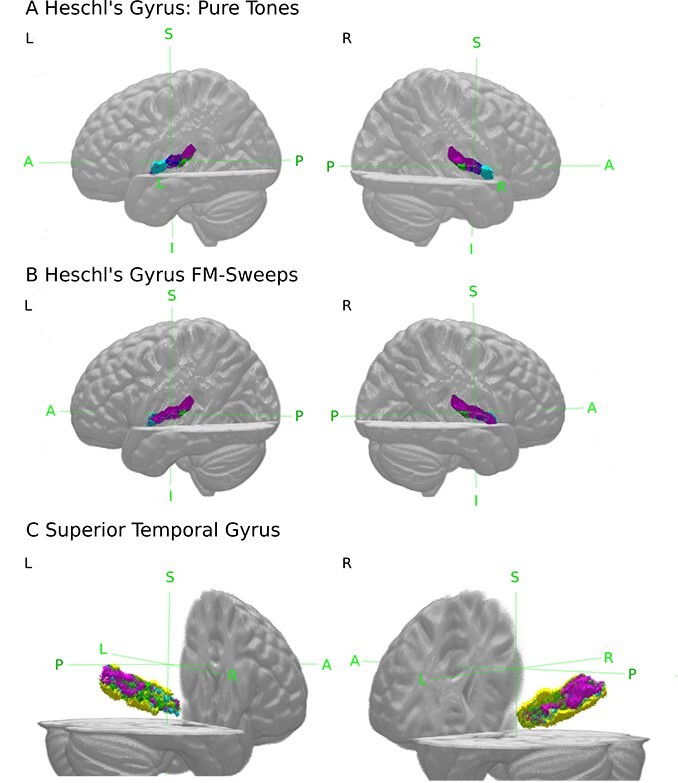
(A and B) 3D view of voxels showing significant ($P < 0.05$, FDR-corrected) SSA (purple) within Te1.0 (dark blue), Te1.1 (green), and Te1.2 (light blue). (C) Voxels of Te3 (yellow) showing significant ($P < 0.05$, FDR-corrected) SSA to both, pure tones and FM-sweeps (purple), voxels showing SSA only to pure tones (blue) or FM-sweeps (green).

For pure tones, SSA voxels were located lateral within bilateral Te1.0, superior within bilateral Te1.1, and predominantly posterior in right Te3 (Fig. 3). For FM-sweeps, the majority of voxels in bilateral Te1.0 and Te1.1 showed significant SSA. SSA voxels in bilateral Te1.2 were localized posterolaterally. In Te3, SSA voxels were mostly found in posterior areas, mirroring the findings from the pure tone experiment (Fig. 3C).

The SSA magnitude $SSAi$ was similarly distributed across all anatomical ROIs and both stimulus types (Fig. 2, lower panels). This indicates that, in opposite to animal literature (Nieto-Diego and Malmierca 2016; Parras et al. 2017), SSA magnitude did not differ between areas of the primary and secondary AC.

### SSA to pure tone and FM-sweep deviants is driven by the encoding of prediction error

To test whether SSA regions encoded prediction error, we examined whether subjective expectations modulated the responses to deviants in different positions by testing the linear relationship between deviant predictability and deviant responses. Generally, beta estimates qualitatively decreased with increasing deviant predictability for both stimulus types in accordance with the predictive coding hypothesis. We corroborated the effect quantitatively by conducting pair-wise statistical comparisons between the responses to each deviant position at the group-level (Supplementary Tables S1 and S2).

In most SSA ROIs, deviants in position four elicited stronger responses than deviants in positions five and six ($P < 0.03$); deviants in position five elicited stronger responses than deviants in position six ($P < 0.04$; all $P$-values of the pure tone data corrected for 30 comparisons and all $P$-values of the FM-sweep data corrected for 48 comparisons).

Responses to deviants in position six were not statistically different from responses to standards after the deviants in most SSA ROIs and both stimulus types, again in consistency with the predictive coding framework. The only exception was Te1.1 R in the pure tone experiment, where responses to deviants in position six were significantly higher than responses to standards after the deviant. In the remaining regions, full predictability seemed to eliminate deviant detection responses completely. It might be tempting to alternatively interpret these results as an effect of selective attention: both deviants in position six and standards after the deviant are irrelevant for the task because the correct response in each trial is already known; while tones in positions four and five are the most relevant for the task because their identity is uncertain. We find this interpretation unconvincing because of two reasons: (i) unattended but unexpected deviants have been shown to elicit stronger responses than unattended standards in a previous experiment with a much lower statistical power (Cacciaglia et al. 2015), but in our data responses to $std2$ were comparable to the responses to $dev6$ (Supplementary Tables S1 and S2) and (ii) deviants in position four and five elicited responses that correlated with their predictability, even though both would putatively engage the same level of attention.

The reported pair-wise differences were further confirmed estimating an LMM relating deviant predictability with their associated BOLD responses at the group-level in each SSA ROI. In line with our hypothesis, we found a significant negative effect of predictability on responses in most SSA ROIs of both stimulus types (Table 3).

**Table 3:** Fixed effect coefficients of the group-level LMM. Pure tone model: beta $\sim $ 1 + predictability + (1 + predictability|run) + (1 + predictability|participant); FM-sweep model: beta $\sim $ 1 + predictability + (1 + predictability|session) + (1 + predictability|run) + (1 + predictability|participant). *Int.*: Intercept; *Pred.*: fixed effect regressor of the model, predictability of the deviants; *DF*: degrees of freedom; $P$: $P$-value of the $t$-test testing for the equality of the coefficient to zero; *CI*: limits of the confidence interval for the coefficients. All $P$-values were Bonferroni-corrected for $N = 13$ SSA ROIs. Empty cells indicate that no SSA-cluster with a statistically significant $P$-value was found in the respective ROIs.

		Pure Tones				FM-Sweeps			
ROI	Name	$\beta $	DF	$P$	CI	$\beta $	DF	$P$	CI
Te1.0 L	Int.	0.89	113770	2.1e-02	[ 0.33, 1.45]	0.87	437596	8.2e-05	[ 0.49, 1.25]
	Pred.	−1.13	113770	4.3e-02	[−1.89, -0.38]	−1.27	437596	3.8e-04	[−1.86, -0.67]
Te1.0 R	Int.	0.10	170542	4.2e-05	[ 0.57, 1.39]	0.80	480583	3.0e-05	[ 0.47, 1.13]
	Pred.	−1.245	170542	3.6e-04	[−1.82, -0.66]	−1.11	480583	1.1e-03	[−1.67, -0.55]
Te1.1 L	Int.	1.22	202234	8.4e-05	[ 0.69, 1.04]	1.04	527434	9.2e-11	[ 0.74, 1.34]
	Pred.	− 1.52	202234	4.9e-04	[−2.24, -0.80]	−1.47	527434	8.5e-11	[−1.89, -1.95]
Te1.1 R	Int.	1.17	312814	1.4e-05	[ 0.70, 1.64]	0.90	682477	1.3e-11	[ 0.65, 1.15]
	Pred.	−1.41	312814	4.6e-04	[−2.08, -0.74]	−1.36	682477	4.2e-10	[−1.77, -0.96]
Te1.2 L	Int.	−	−	−	−	0.69	275308	3.9e-02	[ 0.23, 1.14]
	Pred.	−	−	−	−	−0.96	275308	8.2e-02	[−1.65, -0.27]
Te1.2 R	Int.	−	−	−	−	0.49	256471	6.7e-02	[ 0.14, 0.84]
	Pred.	−	−	−	−	−0.60	256471	3.9e-01	[−1.14, -0.06]
Te3 L	Int.	−	−	−	−	0.61	1732036	1.4e-05	[ 0.36, 0.86]
	Pred.	−	−	−	−	−0.75	1732036	4.4e-04	[−1.11, -0.40]
Te3 R	Int.	0.70	556090	3.8e-02	[ 0.23, 1.16]	0.70	1618531	3.1e-05	[ 0.41, 1.00]
	Pred.	−0.8	556090	1.4e-01	[−1.46, -0.19]	−0.83	1618531	2.6e-03	[−1.26, -0.39]

We could replicate the group-level results in most individual participants for both stimulus types: the correlation between deviant predictability and deviant responses were significantly negative in all but one participant for the pure tone data (for those participants $\rho \in [-0.66, -.011]$, all $P < 10^{-7}$, see Supplementary Fig. S1). For FM-sweeps, this was also the case in 16 out of 18 participants (for these participants $\rho \in [-0.68, -0.11]$, all $P < 10^{-91}$, see Supplementary Fig. S2).

### Neural responses to pure tones and FM-sweeps are best explained by predictive coding

**Fig. 4:**
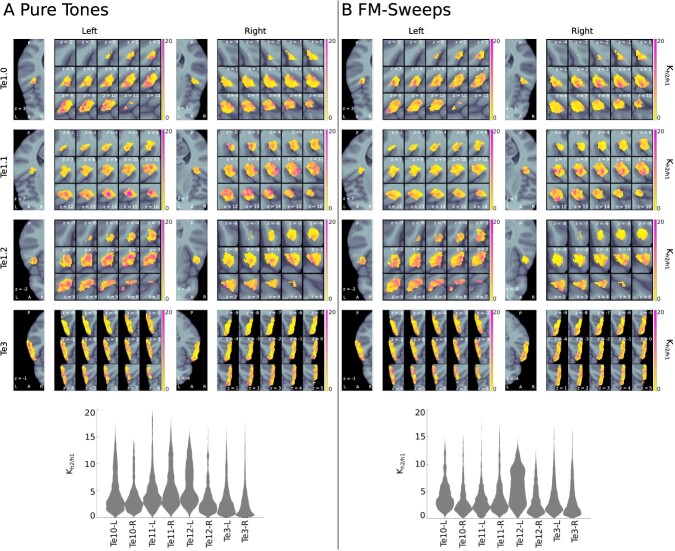
Bayes’ $K_{h2/h1}$ for pure tones and FM-sweeps. The upper panels show $K_{h2/h1}$ in each voxel of the anatomical ROIs; the lower panels show the distribution of $K_{h2/h1}$ across all voxels of the anatomical ROIs. (A) Pure tones; (B) FM-sweeps. The results indicate evidence in favor of the prediction error model (h2) in most voxels of all anatomical ROIs for both stimulus types.

The prediction error model (h2) outperformed the habituation model (h1) in most voxels of all ROIs for both stimulus types, indicating that the effects of predictability are stronger than the effects of habituation in the investigated ROIs. Voxel-wise maps and the distributions of the Bayes factor $K_{h2/h1}$ comparing both models are shown in Fig. 4.

For the pure tone data, the prediction error model (h2) was the best explanation for the data (posterior density of h2 $P> 0.5$) in 96% of voxels in Te1.0 L, 94% in Te1.0 R, 97% in Te1.1 L, 99% in Te1.1 R, 99% in Te1.2 L, 90% in Te1.2 R, 82% in Te3 L, and 72% in Te3 R. H2 was also the best explanation for the FM-sweep data in a majority of voxels of all anatomical ROIs (98% of voxels in Te1.0 L, 89% in Te1.0 R, 90% in Te1.1 L, 94% in Te1.1 R, 97% in Te1.2 L, 83% in Te1.2 R, 91% in Te3 L, and 82% in Te3 R).

## Discussion

The main tenet of the predictive coding framework is that sensory neurons encode prediction error with respect to an internal generative model of the sensory world (Friston 2003; Mumford 1992; Rao and Ballard 1999; Spratling 2017). Despite a growing body of evidence demonstrating prediction error encoding of pure tones in the human AC, many previous studies have potentially confounded habituation with prediction error, disregarded the possibility that prediction error encoding differs across fields within the AC, and focused on the encoding of pure tones, neglecting the rich diversity of dynamic sounds that populates the auditory landscape.

The present study yielded three key findings: first, we found that the prediction error model explained our data better than the habituation model. This suggests that, at least at the coarse temporal resolution of fMRI, BOLD responses to sequences of sounds in the AC were mainly driven by the participants' expectations and that stimulus-specific adaptation (SSA) reflects prediction error as proposed by the predictive coding framework. Second, we showed that the magnitude of SSA was similar across different auditory fields, indicating that there is no systematic difference between adaptation in the primary and secondary auditory pathways in humans. Third, we demonstrated that SSA and prediction error encoding are pervasive in pure tones and frequency-modulated sweeps, suggesting that both stimulus types share a common mechanism for the computation of prediction error. Together, our results indicate that predictive coding is the general encoding mechanism of acoustic information in the human AC.

We found significant SSA to pure tones in bilateral Te1.0 and Te1.1 and in right Te3; SSA in Te1.2 did not reach statistical significance due to a lack of adaptation to the standards. Previous studies have shown that Te1.0 (Bailey et al. 2007) and Te1.2 (Patterson et al. 2002; von Kriegstein et al. 2010) selectively responded to pitch in auditory stimuli, whereas Te1.1 responded selectively to broadband matched noises (Bailey et al. 2007; von Kriegstein et al. 2010). Consequently, Te1.2 has been suggested as the locus of pitch processing in the human AC (De Angelis et al. 2018). Since pitch perception requires integrating auditory information along time windows of only a few milliseconds (Balaguer-Ballester et al. 2008; Hall III and Peters 1981; Meddis and O’Mard 1997), pitch sensitive areas might be insensitive to regularities unfolding at longer time spans (e.g. the ITI of 700 ms that we used for our experiments).

Further work will need to examine how regions that are particularly sensitive to one specific auditory feature show adaptation to other features. For instance, a recent study (An et al. 2021) has shown that evoked responses and mismatch responses to different stimulus features (including pitch) in the rat’s AC show different spatial distributions, with broader distributed mismatch responses.

Our results are the first robust evidence for prediction error encoding of FM in human AC. In line with our results, SSA to FM direction was reported in A1 of rats (Klein et al. 2014). Results from the human literature are more difficult to reconcile with our findings. Three previous studies reported a significant MMN to deviating FM-sweeps (Cornella et al. 2013; Hsieh and Yeh 2021; Kung et al. 2020), but since they did not localize the source of the potentials, it remained unclear to what extent these responses were generated in the AC. One further study investigated sources in the AC but reported no significant results (Altmann et al. 2011). Three other studies reported increasing neuromagnetic responses to repeated FM-sweeps (Heinemann et al. 2011; Heinemann et al. 2010; Okamoto and Kakigi 2017), in direct contradiction with predictive coding. One of these studies (Okamoto and Kakigi 2017) reported the effect specifically in the AC. The short ISIs used in some of these latter studies (e.g. 200 and 100 ms in Heinemann et al. 2010 and Heinemann et al. 2011, respectively) might have contributed to the contradictory results: different temporal integration mechanisms might apply to stimulus sequences spanning shorter or longer time scales.

Our study had a much lower temporal resolution than previous E/MEG studies, with a TR that was longer than twice the ITI. We adopted several strategies to circumvent this problem. First, we ensured that deviants were separated by an average of 5 s, which allowed us to robustly estimate their responses; since inspecting the responses to the deviants is sufficient to differentiate between prediction error and habituation, the temporal resolution did not detriment the potential of the paradigm to answer our main research question. Second, we used eight-tone sequences to ensure that the first standard within each sequence was always sufficiently separated from the previous and the next deviant, also allowing for an accurate estimation of its responses. Estimating the responses to the repeated standards was substantially more challenging; we circumvented this problem by introducing the reasonable assumption that responses to subsequent repeated standards would only vary linearly, allowing us to replace all repetitions with four single parameters. Since the repeated standards are the most common regressor in the design, the GLMs had also more data to accurately estimate their responses. Nevertheless, the comparatively lower temporal resolution of our study should be kept in mind when interpreting our findings.

With respect to SSA magnitude, we did not find differences between primary and secondary auditory areas: magnitudes were comparable across all ROIs. However, the proportion of SSA-voxels was descriptively lower in Te3 compared with all other ROIs for FM-sweeps and compared with Te1.0 and Te1.1 for pure tones. This result suggests that not all auditory fields might exhibit SSA to the same extent. Furthermore, we found deviant detection and repetition suppression for pure tones to differ across fields: Te1.2 was dominated by deviant detection, while all other ROIs additionally showed significant repetition suppression and thus SSA.

In rodents, on the contrary, SSA magnitude clearly differed between fields of the primary and secondary auditory pathway in previous studies: secondary cortical areas showed stronger SSA than primary cortical areas (Nieto-Diego and Malmierca 2016; Parras et al. 2017) and the same pattern was reported in the subcortical auditory pathway (Antunes and Malmierca 2011; Antunes et al. 2010; Ayala et al. 2015; Duque et al. 2014; Duque et al. 2012; Parras et al. 2017).

The discrepancies between studies in rodents and our results may stem from a poor correspondence between auditory fields in these two species: while AC in rodents is typically subdivided in three lemniscal and two nonlemniscal fields (Parras et al. 2017), in primates, AC is subdivided in three distinct fields: core, belt, and parabelt (Besle et al. 2019). We used a previous segmentation of human AC with four different fields (Morosan et al. 2001) that cannot be trivially mapped to the animal subdivisions and whose classification as primary or secondary is still of debate (Besle et al. 2019; Moerel et al. 2014). It is possible that using a different anatomical segmentation (e.g. using Brodman areas instead) would yield different results. However, our previous results showed that the distributions of SSA in the subcortical auditory pathway, which is closely replicated across mammals (Glendenning and Masterton 1998), also differed between humans and rodents: SSA magnitude in our human subjects was similar across different primary and secondary subcortical auditory areas (Tabas et al. 2021b; Tabas et al. 2020). Moreover, the spatial and temporal resolution of fMRI might obscure differences between auditory fields that may only be apparent in single-unit recordings.

Until recently, it was unclear whether predictions from generative model units inform prediction errors only at the immediate lower stage of the processing hierarchy or also at subsequently lower stages (see Tabas et al. 2021a for a review of the empirical evidence on both standpoints). MMN studies showed that prediction error is elicited with respect to high-level expectations; namely by the violation of complex statistical regularities (see Paavilainen 2013 for review), the omission of expected sounds (Bendixen et al. 2009; Chennu et al. 2016; Wacongne et al. 2011), and abstract expectations about the occurrence of deviating sounds (Wacongne et al. 2011). However, since the generators of the MMN are partly located in the frontal cortex (Paavilainen 2013), MMN research cannot clarify whether subjective expectations are used to compute prediction errors at lower levels of the auditory processing hierarchy.

We found prediction error encoding to be a dominant encoding principle for both stimulus types in all anatomical ROIs. This result suggests that high-level predictions informed by the task instructions, putatively computed in regions at higher processing stages than the sensory cortices, are used to compute prediction errors in the primary AC. We had previously shown that these same predictions are also used to compute prediction error in the human auditory midbrain and thalamus (Tabas et al. 2021b; Tabas et al. 2020). Previous studies also showed that prediction error in the AC was computed with respect to language-specific expectations (e.g. Blank and Davis 2016; Blank et al. 2018; Heilbron et al. 2021; Ylinen et al. 2016). Together, the empirical evidence supports the hypothesis that high-level predictions are used to compute prediction errors along the entire predictive processing hierarchy.

Our study did not tackle the question of where SSA and prediction errors are first generated. Since auditory cortical areas receive direct bottom-up input from the auditory thalamus (Schofield 2011), SSA and prediction error signals in AC could reflect ascending input from prediction error units in subcortical structures (Tabas et al. 2021b; Tabas et al. 2020). Conversely, subcortical SSA and prediction error signals might as well be inherited from cerebral cortex areas via corticofugal modulation (Malmierca 2015). Animal studies have shown that SSA in secondary auditory midbrain and thalamus persists under deactivation of the AC (Anderson and Malmierca 2013; Antunes and Malmierca 2011), but not in the primary auditory thalamus, where SSA is the weakest (Bäuerle et al. 2011). This suggests that subcortical SSA cannot be entirely inherited from the AC. Further work is needed to clarify the interplay of bottom-up and top-down signaling in the computation of prediction error.

Our results suggest that predictive coding is the general mechanism underlying the encoding of acoustic features in AC. Impaired predictive processes in AC have been linked to speech processing disorders and clinical conditions such as developmental dyslexia (e.g. Gu and Bi 2020; Neuhoff et al. 2012; Plakas et al. 2013), stuttering (Daliri and Max 2015), autism spectrum disorder (van Schalkwyk et al. 2017), psychosis (Fryer et al. 2020; Sterzer et al. 2018), or schizophrenia (Perez et al. 2014). Investigating how predictive coding is implemented in the human AC is essential for a mechanistic understanding of perception and its dysfunction.

## Supplementary Material

SupplementaryMaterial_PredictiveEncodingOfPureTonesAndFMSweepsInTheHumanAuditoryCortex_tgac047

## References

[ref1] Altmann CF , Gaese BH. Representation of frequency-modulated sounds in the human brain. Hear Res. 2014:307:74–85. 23933098 10.1016/j.heares.2013.07.018

[ref2] Altmann CF , Klein C, Heinemann LV, Wibral M, Gaese BH, Kaiser J. Repetition of complex frequency-modulated sweeps enhances neuromagnetic responses in the human auditory cortex. Hear Res. 2011:282(1–2):216–224. 21839158 10.1016/j.heares.2011.07.008

[ref3] An H , Auksztulewicz R, Kang H, Schnupp JW. Cortical mapping of mismatch responses to independent acoustic features. Hear Res. 2021:399:107894. 10.1016/j.heares.2020.107894. 31987647 PMC8354208

[ref4] Anderson L , Malmierca M. The effect of auditory cortex deactivation on stimulus-specific adaptation in the inferior colliculus of the rat. Eur J Neurosci. 2013:37(1):52–62. 23121128 10.1111/ejn.12018

[ref5] Antunes FM , Malmierca MS. Effect of auditory cortex deactivation on stimulus-specific adaptation in the medial geniculate body. J Neurosci. 2011:31(47):17306–17316. 22114297 10.1523/JNEUROSCI.1915-11.2011 PMC6623836

[ref6] Antunes FM , Nelken I, Covey E, Malmierca MS. Stimulus-specific adaptation in the auditory thalamus of the anesthetized rat. PLoS One. 2010:5(11):e14071. 10.1371/journal.pone.0014071. 21124913 PMC2988819

[ref7] Avants BB , Tustison NJ, Song G, Cook PA, Klein A, Gee JC. A reproducible evaluation of ants similarity metric performance in brain image registration. NeuroImage. 2011:54(3):2033–2044. 20851191 10.1016/j.neuroimage.2010.09.025 PMC3065962

[ref8] Ayala YA , Udeh A, Dutta K, Bishop D, Malmierca MS, Oliver DL. Differences in the strength of cortical and brainstem inputs to ssa and non-ssa neurons in the inferior colliculus. Sci Rep. 2015:5(1):1–17. 10.1038/srep10383 PMC4438612 25993334

[ref9] Bailey L , Abolmaesumi P, Tam J, Morosan P, Cusack R, Amunts K, Johnsrude I. Customised cytoarchitectonic probability maps using deformable registration: primary auditory cortex. In: International Conference on Medical Image Computing and Computer-Assisted Intervention. Berlin: Springer; 2007. pp. 760–768. 10.1007/978-3-540-75759-7_92 18044637

[ref10] Balaguer-Ballester E , Denham SL, Meddis R. A cascade autocorrelation model of pitch perception. J Acoust Soc Am. 2008:124(4):2186–2195. 19062858 10.1121/1.2967829

[ref11] Bäuerle P , von der Behrens W, Kössl M, Gaese BH. Stimulus-specific adaptation in the gerbil primary auditory thalamus is the result of a fast frequency-specific habituation and is regulated by the corticofugal system. J Neurosci. 2011:31(26):9708–9722. 21715636 10.1523/JNEUROSCI.5814-10.2011 PMC6623171

[ref12] Bendixen A , Schröger E, Winkler I. I heard that coming: event-related potential evidence for stimulus-driven prediction in the auditory system. J Neurosci. 2009:29(26):8447–8451. 19571135 10.1523/JNEUROSCI.1493-09.2009 PMC6665649

[ref13] Benjamini Y , Hochberg Y. Controlling the false discovery rate: a practical and powerful approach to multiple testing. J R Stat Soc Ser B Methodol. 1995:57(1):289–300.

[ref14] Berlot E , Formisano E, De Martino F. Mapping frequency-specific tone predictions in the human auditory cortex at high spatial resolution. J Neurosci. 2018:38(21):4934–4942. 29712781 10.1523/JNEUROSCI.2205-17.2018 PMC6596130

[ref15] Besle J , Mougin O, Sánchez-Panchuelo R-M, Lanting C, Gowland P, Bowtell R, Francis S, Krumbholz K. Is human auditory cortex organization compatible with the monkey model? contrary evidence from ultra-high-field functional and structural mri. Cereb Cortex. 2019:29(1):410–428. 30357410 10.1093/cercor/bhy267 PMC6294415

[ref16] Blank H , Davis MH. Prediction errors but not sharpened signals simulate multivoxel fmri patterns during speech perception. PLoS Biol. 2016:14(11):e1002577. 10.1371/journal.pbio.1002577. 27846209 PMC5112801

[ref17] Blank H , Spangenberg M, Davis MH. Neural prediction errors distinguish perception and misperception of speech. J Neurosci. 2018:38(27):6076–6089. 29891730 10.1523/JNEUROSCI.3258-17.2018 PMC6596154

[ref18] Cacciaglia R , Escera C, Slabu L, Grimm S, Sanjuán A, Ventura-Campos N, Ávila C. Involvement of the human midbrain and thalamus in auditory deviance detection. Neuropsychologia. 2015:68:51–58. 25556848 10.1016/j.neuropsychologia.2015.01.001

[ref19] Cacciaglia R , Costa-Faidella J, Zarnowiec K, Grimm S, Escera C. Auditory predictions shape the neural responses to stimulus repetition and sensory change. NeuroImage. 2019:186:200–210. 30414982 10.1016/j.neuroimage.2018.11.007

[ref20] Carbajal GV , Malmierca MS. The neuronal basis of predictive coding along the auditory pathway: from the subcortical roots to cortical deviance detection. Trends Hear. 2018:22:1–33. 10.1177/2331216518784822 PMC6053868 30022729

[ref21] Chalk M , Seitz AR, Seriès P. Rapidly learned stimulus expectations alter perception of motion. J Vis. 2010:10(8):2:1–18. 10.1167/10.8.2 20884577

[ref22] Chambers C , Akram S, Adam V, Pelofi C, Sahani M, Shamma S, Pressnitzer D. Prior context in audition informs binding and shapes simple features. Nat Commun. 2017:8(1):1–11. 28425433 10.1038/ncomms15027 PMC5411480

[ref23] Chennu S , Noreika V, Gueorguiev D, Shtyrov Y, Bekinschtein TA, Henson R. Silent expectations: dynamic causal modeling of cortical prediction and attention to sounds that weren’t. J Neurosci. 2016:36(32):8305–8316. 27511005 10.1523/JNEUROSCI.1125-16.2016 PMC4978796

[ref24] Cornella M , Leung S, Grimm S, Escera C. Detection of simple and pattern regularity violations occurs at different levels of the auditory hierarchy. PLoS One. 2012:7(8):e43604. 10.1371/journal.pone.0043604. 22916282 PMC3423368

[ref25] Cornella M , Leung S, Grimm S, Escera C. Regularity encoding and deviance detection of frequency modulated sweeps: Human middle-and long-latency auditory evoked potentials. Psychophysiology. 2013:50(12):1275–1281. 24016075 10.1111/psyp.12137

[ref26] Costa-Faidella J , Baldeweg T, Grimm S, Escera C. Interactions between “what” and “when” in the auditory system: temporal predictability enhances repetition suppression. J Neurosci. 2011:31(50):18590–18597. 22171057 10.1523/JNEUROSCI.2599-11.2011 PMC6623902

[ref27] Daliri A , Max L. Electrophysiological evidence for a general auditory prediction deficit in adults who stutter. Brain Lang. 2015:150:37–44. 26335995 10.1016/j.bandl.2015.08.008 PMC4663101

[ref28] De Angelis V , De Martino F, Moerel M, Santoro R, Hausfeld L, Formisano E. Cortical processing of pitch: Model-based encoding and decoding of auditory fmri responses to real-life sounds. NeuroImage. 2018:180:291–300. 29146377 10.1016/j.neuroimage.2017.11.020

[ref29] De Lange FP , Heilbron M, Kok P. How do expectations shape perception? Trends Cogn Sci. 2018:22(9):764–779. 30122170 10.1016/j.tics.2018.06.002

[ref30] Deouell LY . The frontal generator of the mismatch negativity revisited. J Psychophysiol. 2007:21(3–4):188–203.

[ref31] Divenyi P . Perception of complete and incomplete formant transitions in vowels. J Acoust Soc Am. 2009:126(3):1427–1439. 19739756 10.1121/1.3167482 PMC2809691

[ref32] Duque D , Pérez-González D, Ayala YA, Palmer AR, Malmierca MS. Topographic distribution, frequency, and intensity dependence of stimulus-specific adaptation in the inferior colliculus of the rat. J Neurosci. 2012:32(49):17762–17774. 23223296 10.1523/JNEUROSCI.3190-12.2012 PMC6621662

[ref33] Duque D , Malmierca MS, Caspary DM. Modulation of stimulus-specific adaptation by gabaa receptor activation or blockade in the medial geniculate body of the anaesthetized rat. J Physiol. 2014:592(4):729–743. 24099802 10.1113/jphysiol.2013.261941 PMC3934711

[ref34] Dürschmid S , Edwards E, Reichert C, Dewar C, Hinrichs H, Heinze H-J, Kirsch HE, Dalal SS, Deouell LY, Knight RT. Hierarchy of prediction errors for auditory events in human temporal and frontal cortex. Proc Natl Acad Sci. 2016:113(24):6755–6760. 27247381 10.1073/pnas.1525030113 PMC4914143

[ref35] Eytan D , Brenner N, Marom S. Selective adaptation in networks of cortical neurons. J Neurosci. 2003:23(28):9349–9356. 14561862 10.1523/JNEUROSCI.23-28-09349.2003 PMC6740578

[ref36] Fischl B , Salat D, Busa E, Albert M, Dieterich M, Haselgrove C. Whole brain segmentation: automated labeling of neuroanatomical structures in the human brain. Neuron. 2002:33(3):341–355. 11832223 10.1016/s0896-6273(02)00569-x

[ref37] Friston K . Learning and inference in the brain. Neural Netw. 2003:16(9):1325–1352. 14622888 10.1016/j.neunet.2003.06.005

[ref38] Friston K , Zarahn E, Josephs O, Henson RN, Dale AM. Stochastic designs in event-related fmri. NeuroImage. 1999:10(5):607–619. 10547338 10.1006/nimg.1999.0498

[ref39] Fryer SL , Roach BJ, Hamilton HK, Bachman P, Belger A, Carrión RE, Duncan E, Johannesen J, Light GA, Niznikiewicz M, et al. Deficits in auditory predictive coding in individuals with the psychosis risk syndrome: Prediction of conversion to psychosis. J Abnorm Psychol. 2020:129(6):599–611. 32757603 10.1037/abn0000513 PMC7430496

[ref40] Geis R , Borst G. Intracellular responses to frequency modulated tones in the dorsal cortex of the mouse inferior colliculus. Front Neural Circuits. 2013:7:7. 10.3389/fncir.2013.00007. 23386812 PMC3560375

[ref41] Glendenning K , Masterton R. Comparative morphometry of mammalian central auditory systems: variation in nuclei and form of the ascending system. Brain Behav Evol. 1998:51(2):59–89. 9491274 10.1159/000006530

[ref42] Gorgolewski K , Burns CD, Madison C, Clark D, Halchenko YO, Waskom ML, Ghosh SS. Nipype: a flexible, lightweight and extensible neuroimaging data processing framework in python. Front Neuroinform. 2011:5:13. 10.3389/fninf.2011.00013. 21897815 PMC3159964

[ref43] Gu C , Bi H-Y. Auditory processing deficit in individuals with dyslexia: A meta-analysis of mismatch negativity. Neurosci Biobehav Rev. 2020:116:396–405. 10.1016/j.neubiorev.2020.06.032 32610180

[ref44] Gulban OF , Goebel R, Moerel M, Zachlod D, Mohlberg H, Amunts K, De Martino F. Improving a probabilistic cytoarchitectonic atlas of auditory cortex using a novel method for inter-individual alignment. elife. 2020:9:e56963. 10.7554/eLife.56963. 32755545 PMC7406353

[ref45] Hackett TA , Preuss TM, Kaas JH. Architectonic identification of the core region in auditory cortex of macaques, chimpanzees, and humans. J Comp Neurol. 2001:441(3):197–222. 11745645 10.1002/cne.1407

[ref46] Hall JW III , Peters RW. Pitch for nonsimultaneous successive harmonics in quiet and noise. The Journal of the Acoustical Society of America. 1981:69(2):509–513. 7462473 10.1121/1.385480

[ref47] Hall DA , Haggard MP, Akeroyd MA, Summerfield AQ, Palmer AR, Elliott MR, Bowtell RW. Modulation and task effects in auditory processing measured using fmri. Hum Brain Mapp. 2000:10(3):107–119. 10912590 10.1002/1097-0193(200007)10:3<107::AID-HBM20>3.0.CO;2-8 PMC6871907

[ref48] Hart HC , Palmer AR, Hall DA. Amplitude and frequency-modulated stimuli activate common regions of human auditory cortex. Cereb Cortex. 2003:13(7):773–781. 12816893 10.1093/cercor/13.7.773

[ref49] Heilbron M , Armeni K, Schoffelen J-M, Hagoort P, de Lange FP, A hierarchy of linguistic predictions during natural language comprehension. Proceedings of the National Academy of Sciences. 2022:119(32):e2201968119. 10.1073/pnas.2201968119. PMC9371745 35921434

[ref50] Heinemann LV , Rahm B, Kaiser J, Gaese BH, Altmann CF. Repetition enhancement for frequency-modulated but not unmodulated sounds: a human meg study. PLoS One. 2010:5(12):e15548. 21217825 10.1371/journal.pone.0015548 PMC3013102

[ref51] Heinemann LV , Kaiser J, Altmann CF. Auditory repetition enhancement at short interstimulus intervals for frequency-modulated tones. Brain Res. 2011:1411:65–75. 21803340 10.1016/j.brainres.2011.07.009

[ref52] Holm S . A simple sequentially rejective multiple test procedure. Scand J Stat. 1979:6(2):65–70.

[ref53] Hsieh I-H , Yeh W-T. The interaction between timescale and pitch contour at pre-attentive processing of frequency-modulated sweeps. Front Psychol. 2021:12:697. 10.3389/fpsyg.2021.637289. PMC8021897 33833720

[ref54] Hu B . Functional organization of lemniscal and nonlemniscal auditory thalamus. Exp Brain Res. 2003:153(4):543–549. 12937877 10.1007/s00221-003-1611-5

[ref55] Issa JB , Haeffele BD, Young ED, Yue DT. Multiscale mapping of frequency sweep rate in mouse auditory cortex. Hear Res. 2017:344:207–222. 28011084 10.1016/j.heares.2016.11.018 PMC5240629

[ref56] Jenkinson M , Beckmann CF, Behrens TE, Woolrich MW, Smith SM. Fsl. NeuroImage. 2012:62(2):782–790. 21979382 10.1016/j.neuroimage.2011.09.015

[ref57] Klein C , von der Behrens W, Gaese BH. Stimulus-specific adaptation in field potentials and neuronal responses to frequency-modulated tones in the primary auditory cortex. Brain Topogr. 2014:27(4):599–610. 24863565 10.1007/s10548-014-0376-4

[ref58] von Kriegstein K , Smith DR, Patterson RD, Kiebel SJ, Griffiths TD. How the human brain recognizes speech in the context of changing speakers. J Neurosci. 2010:30(2):629–638. 20071527 10.1523/JNEUROSCI.2742-09.2010 PMC2824128

[ref59] Kung S-J , Wu DH, Hsu C-H, Hsieh I-H. A minimum temporal window for direction detection of frequency-modulated sweeps: A magnetoencephalography study. Front Psychol. 2020:11:389. 32218758 10.3389/fpsyg.2020.00389 PMC7078663

[ref60] Lange K . Brain correlates of early auditory processing are attenuated by expectations for time and pitch. Brain Cogn. 2009:69(1):127–137. 18644669 10.1016/j.bandc.2008.06.004

[ref61] Lecaignard F , Bertrand O, Gimenez G, Mattout J, Caclin A. Implicit learning of predictable sound sequences modulates human brain responses at different levels of the auditory hierarchy. Front Hum Neurosci. 2015:9:505. 10.3389/fnhum.2015.00505. 26441602 PMC4584941

[ref62] Lee CC , Sherman SM. On the classification of pathways in the auditory midbrain, thalamus, and cortex. Hear Res. 2011:276(1–2):79–87. 21184817 10.1016/j.heares.2010.12.012 PMC3108009

[ref63] Leonard MK , Baud MO, Sjerps MJ, Chang EF. Perceptual restoration of masked speech in human cortex. Nat Commun. 2016:7(1):1–9. 10.1038/ncomms13619 PMC5187421 27996973

[ref64] Liberman AM , Cooper FS, Shankweiler DP, Studdert-Kennedy M. Perception of the speech code. Psychol Rev. 1967:74(6):431–461. 4170865 10.1037/h0020279

[ref65] Lui B , Mendelson J. Frequency modulated sweep responses in the medial geniculate nucleus. Exp Brain Res. 2003:153(4):550–553. 12961056 10.1007/s00221-003-1618-y

[ref66] Malmierca MS . Auditory system. In: The rat nervous system. Elsevier; 2015. pp. 865–946.

[ref67] Malmierca MS , Hackett TA. Structural organization of the ascending auditory pathway. In: The Oxford handbook of auditory science: The Auditory Brain. Oxford University Press; 2010. pp. 9–41.

[ref68] Meddis R , O’Mard L. A unitary model of pitch perception. J Acoust Soc Am. 1997:102(3):1811–1820. 9301058 10.1121/1.420088

[ref69] Mill R , Coath M, Wennekers T, Denham SL. A neurocomputational model of stimulus-specific adaptation to oddball and markov sequences. PLoS Comput Biol. 2011:7(8):e1002117. 10.1371/journal.pcbi.1002117. 21876661 PMC3158038

[ref70] Moerel M , De Martino F, Formisano E. An anatomical and functional topography of human auditory cortical areas. Front Neurosci. 2014:8:225. 10.3389/fnins.2014.00225. 25120426 PMC4114190

[ref71] Morosan P , Rademacher J, Schleicher A, Amunts K, Schormann T, Zilles K. Human primary auditory cortex: cytoarchitectonic subdivisions and mapping into a spatial reference system. NeuroImage. 2001:13(4):684–701. 11305897 10.1006/nimg.2000.0715

[ref72] Mumford D . On the computational architecture of the neocortex. Biol Cybern. 1992:66(3):241–251. 1540675 10.1007/BF00198477

[ref73] Neuhoff N , Bruder J, Bartling J, Warnke A, Remschmidt H, Müller-Myhsok B, Schulte-Körne G. Evidence for the late mmn as a neurophysiological endophenotype for dyslexia. PLoS One. 2012:7(5):e34909. 10.1371/journal.pone.0034909. 22606227 PMC3351484

[ref74] Nieto-Diego J , Malmierca MS. Topographic distribution of stimulus-specific adaptation across auditory cortical fields in the anesthetized rat. PLoS Biol. 2016:14(3):e1002397. 10.1371/journal.pbio.1002397. 26950883 PMC4780834

[ref75] Okamoto H , Kakigi R. Modulation of auditory evoked magnetic fields elicited by successive frequency-modulated (fm) sweeps. Front Hum Neurosci. 2017:11:36. 10.3389/fnhum.2017.00036. 28220066 PMC5292620

[ref76] Paavilainen P . The mismatch-negativity (mmn) component of the auditory event-related potential to violations of abstract regularities: a review. Int J Psychophysiol. 2013:88(2):109–123. 23542165 10.1016/j.ijpsycho.2013.03.015

[ref77] Paltoglou AE , Sumner CJ, Hall DA. Mapping feature-sensitivity and attentional modulation in human auditory cortex with functional magnetic resonance imaging. Eur J Neurosci. 2011:33(9):1733–1741. 21447093 10.1111/j.1460-9568.2011.07656.x PMC3110306

[ref78] Parras GG , Nieto-Diego J, Carbajal GV, Valdés-Baizabal C, Escera C, Malmierca MS. Neurons along the auditory pathway exhibit a hierarchical organization of prediction error. Nat Commun. 2017:8(1):1–17. 29247159 10.1038/s41467-017-02038-6 PMC5732270

[ref79] Patterson RD , Uppenkamp S, Johnsrude IS, Griffiths TD. The processing of temporal pitch and melody information in auditory cortex. Neuron. 2002:36(4):767–776. 12441063 10.1016/s0896-6273(02)01060-7

[ref80] Penny WD , Friston KJ, Ashburner JT, Kiebel SJ, Nichols TE. Statistical parametric mapping: the analysis of functional brain images. London: Elsevier; 2011.

[ref81] Perez VB , Woods SW, Roach BJ, Ford JM, McGlashan TH, Srihari VH, Mathalon DH. Automatic auditory processing deficits in schizophrenia and clinical high-risk patients: forecasting psychosis risk with mismatch negativity. Biol Psychiatry. 2014:75(6):459–469. 24050720 10.1016/j.biopsych.2013.07.038 PMC4028131

[ref82] Pérez-González D , Parras GG, Morado-Díaz CJ, Aedo-Sánchez C, Carbajal GV, Malmierca MS. Deviance detection in physiologically identified cell types in the rat auditory cortex. Hear Res. 2021:399:107997. 10.1016/j.heares.2020.107997. 32482383

[ref83] Phillips HN , Blenkmann A, Hughes LE, Kochen S, Bekinschtein TA, Rowe JB, Cam-CAN. Convergent evidence for hierarchical prediction networks from human electrocorticography and magnetoencephalography. Cortex. 2016:82:192–205. 27389803 10.1016/j.cortex.2016.05.001 PMC4981429

[ref84] Plakas A , van Zuijen T, van Leeuwen T, Thomson JM, van der Leij A. Impaired non-speech auditory processing at a pre-reading age is a risk-factor for dyslexia but not a predictor: an erp study. Cortex. 2013:49(4):1034–1045. 22542727 10.1016/j.cortex.2012.02.013

[ref85] Rao RP , Ballard DH. Predictive coding in the visual cortex: a functional interpretation of some extra-classical receptive-field effects. Nat Neurosci. 1999:2(1):79–87. 10195184 10.1038/4580

[ref86] Rosa MJ , Bestmann S, Harrison L, Penny W. Bayesian model selection maps for group studies. NeuroImage. 2010:49(1):217–224. 19732837 10.1016/j.neuroimage.2009.08.051 PMC2791519

[ref87] Rubin J , Ulanovsky N, Nelken I, Tishby N. The representation of prediction error in auditory cortex. PLoS Comput Biol. 2016:12(8):e1005058. 10.1371/journal.pcbi.1005058. 27490251 PMC4973877

[ref89] Schofield BR . Central descending auditory pathways. In: Auditory and vestibular efferents. New York, NY: Springer; 2011. pp. 261–290.

[ref90] Shalgi S , Deouell LY. Direct evidence for differential roles of temporal and frontal components of auditory change detection. Neuropsychologia. 2007:45(8):1878–1888. 17239410 10.1016/j.neuropsychologia.2006.11.023

[ref91] Spratling MW . A review of predictive coding algorithms. Brain Cogn. 2017:112:92–97. 26809759 10.1016/j.bandc.2015.11.003

[ref92] Stephan KE , Penny WD, Daunizeau J, Moran RJ, Friston KJ. Bayesian model selection for group studies. NeuroImage. 2009:46(4):1004–1017. 19306932 10.1016/j.neuroimage.2009.03.025 PMC2703732

[ref93] Sterzer P , Frith C, Petrovic P. Believing is seeing: expectations alter visual awareness. Curr Biol. 2008:18(16):R697–R698. 18727901 10.1016/j.cub.2008.06.021

[ref94] Sterzer P , Adams RA, Fletcher P, Frith C, Lawrie SM, Muckli L, Petrovic P, Uhlhaas P, Voss M, Corlett PR. The predictive coding account of psychosis. Biol Psychiatry. 2018:84(9):634–643. 30007575 10.1016/j.biopsych.2018.05.015 PMC6169400

[ref95] Suga N . Basic acoustic patterns and neural mechanisms shared by humans and animals for auditory perception. In: Listening to speech. New York, NY: Psychology Press; 2012.

[ref96] Tabas A , Mihai G, Kiebel S, Trampel R, von Kriegstein K. Abstract rules drive adaptation in the subcortical sensory pathway. elife. 2020:9:e64501. 10.7554/eLife.64501. 33289479 PMC7785290

[ref97] Tabas A , Kiebel S, Marxen M, von Kriegstein K. Fast frequency modulation is encoded according to the listener expectations in the human subcortical auditory pathway. arXiv preprint arXiv:2108.02066. 2021a. 10.1162/imag_a_00292 PMC12290582 40800274

[ref98] Tabas A , von Kriegstein K. Adjudicating between local and global architectures of predictive processing in the subcortical auditory pathway. Front Neural Circuits. 2021b:15:644743. 10.3389/fncir.2021.644743. 33776657 PMC7994860

[ref99] Todorovic A , de Lange FP. Repetition suppression and expectation suppression are dissociable in time in early auditory evoked fields. J Neurosci. 2012:32(39):13389–13395. 23015429 10.1523/JNEUROSCI.2227-12.2012 PMC6621367

[ref100] Todorovic A , van Ede F, Maris E, de Lange FP. Prior expectation mediates neural adaptation to repeated sounds in the auditory cortex: an meg study. J Neurosci. 2011:31(25):9118–9123. 21697363 10.1523/JNEUROSCI.1425-11.2011 PMC6623501

[ref101] Ulanovsky N , Las L, Nelken I. Processing of low-probability sounds by cortical neurons. Nat Neurosci. 2003:6(4):391–398. 12652303 10.1038/nn1032

[ref102] Ulanovsky N , Las L, Farkas D, Nelken I. Multiple time scales of adaptation in auditory cortex neurons. J Neurosci. 2004:24(46):10440–10453. 15548659 10.1523/JNEUROSCI.1905-04.2004 PMC6730303

[ref88] van Schalkwyk GI , Volkmar FR, Corlett PR. A predictive coding account of psychotic symptoms in autism spectrum disorder. J Autism Dev Disord. 2017:47(5):1323–1340. 28185044 10.1007/s10803-017-3065-9

[ref103] Wacongne C , Labyt E, van Wassenhove V, Bekinschtein T, Naccache L, Dehaene S. Evidence for a hierarchy of predictions and prediction errors in human cortex. Proc Natl Acad Sci. 2011:108(51):20754–20759. 22147913 10.1073/pnas.1117807108 PMC3251061

[ref104] Wang H , Han Y-F, Chan Y-S, He J. Stimulus-specific adaptation at the synapse level in vitro. PLoS One. 2014:9(12):e114537. 10.1371/journal.pone.0114537. 25486252 PMC4259350

[ref105] Ylinen S , Huuskonen M, Mikkola K, Saure E, Sinkkonen T, Paavilainen P. Predictive coding of phonological rules in auditory cortex: A mismatch negativity study. Brain Lang. 2016:162:72–80. 27588355 10.1016/j.bandl.2016.08.007

