# Supplementary material for: Predictive encoding of pure tones and FM-sweeps in the human auditory cortex
Source: Cereb Cortex Commun. 2022 Nov 16;3(4):tgac047. doi: 10.1093/texcom/tgac047 (PMC9764222; doi:10.1093/texcom/tgac047)
Supplement: SupplementaryMaterial_PredictiveEncodingOfPureTonesAndFMSweepsInTheHumanAuditoryCortex_tgac047 [file supplementarymaterial_predictiveencodingofpuretonesandfmsweepsinthehumanauditorycortex_tgac047.pdf]

Supplementary Material

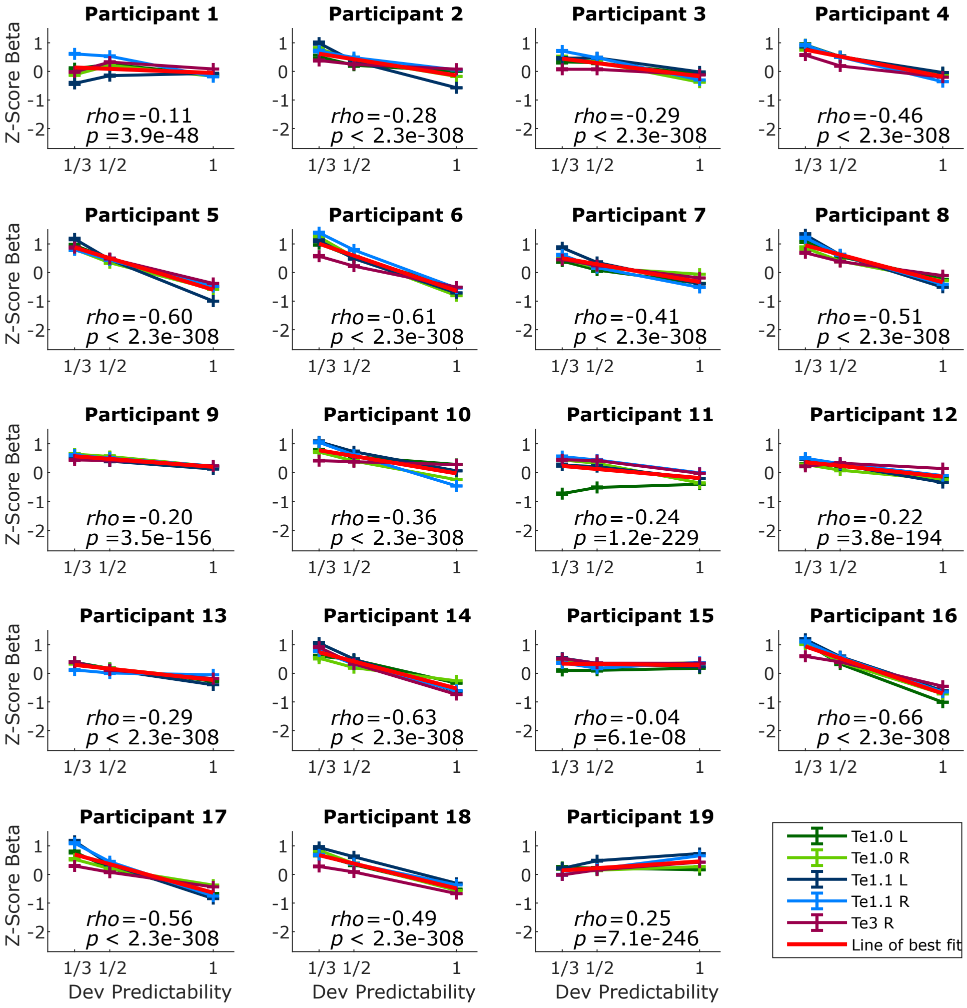

Figure S1: **Spearman's rank correlation between deviant predictability and standardized beta estimates for each participant of the pure tone experiment.** Deviant predictability is shown on the x-axis (1/3 for deviants in position four, 1/2 for deviants in position five, and 1 for deviants in position six). The y-axis shows the respective mean standardized beta estimates.

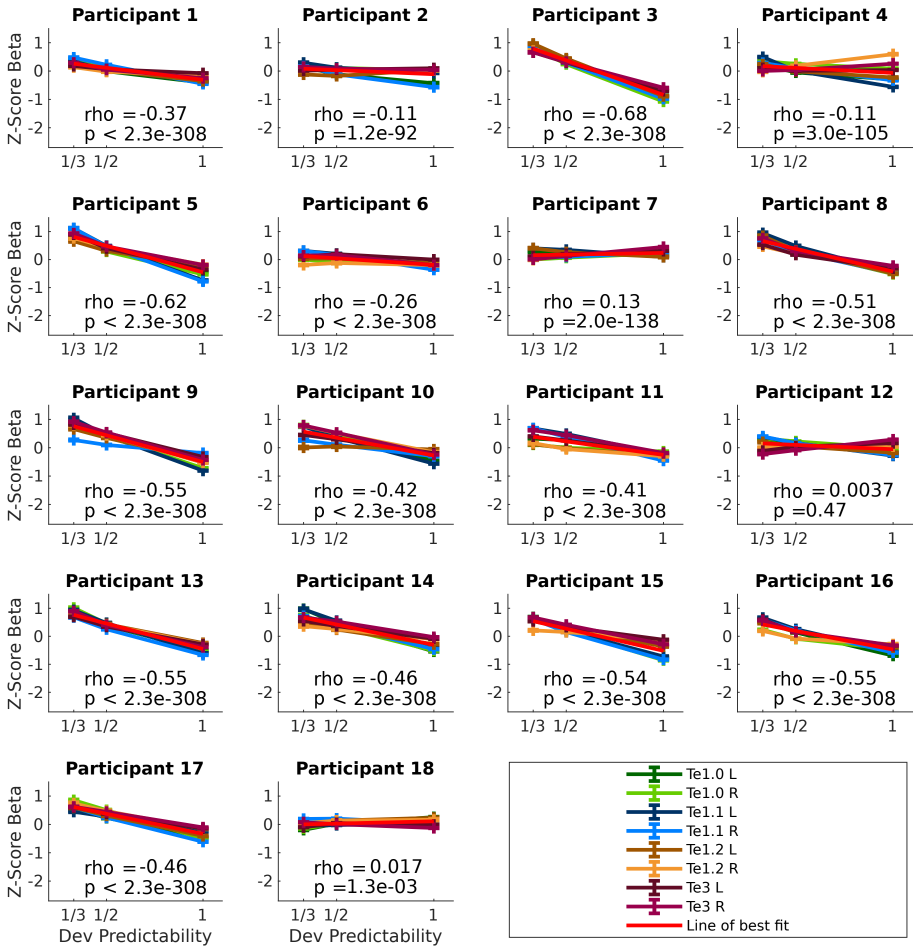

Figure S2: **Spearman's rank correlation between deviant predictability and standardized beta estimates for each participant of the FM-sweep experiment.** Deviant predictability is shown on the x-axis (1/3 for deviants in position four, 1/2 for deviants in position five, and 1 for deviants in position six). The y-axis shows the respective mean standardized beta estimates. The last panel shows the distribution of the correlation coefficient per SSA ROI.

Hypothesis	Te1.0 L	Te1.0 R	Te1.1 L	Te1.1 R	Te3 R
$std0 > std2$	$p = 0.0024$<br>$d = 1.93$	$p = 0.0029$<br>$d = 2.66$	$p = 0.0026$<br>$d = 2.78$	$p = 0.0025$<br>$d = 2.68$	$p = 0.0024$<br>$d = 2.77$
$dev4 > std2$	$p = 0.0047$<br>$d = 2.19$	$p = 0.0023$<br>$d = 3.51$	$p = 0.0031$<br>$d = 3.17$	$p = 0.0021$<br>$d = 3.93$	$p = 0.0021$<br>$d = 3.20$
$dev4 > dev6$	$p = 0.0030$<br>$d = 1.98$	$p = 0.0028$<br>$d = 2.71$	$p = 0.0037$<br>$d = 2.39$	$p = 0.0035$<br>$d = 2.64$	$p = 0.0037$<br>$d = 1.83$
$dev4 > dev5$	$p = 0.0063$<br>$d = 0.67$	$p = 0.0039$<br>$d = 1.14$	$p = 0.0055$<br>$d = 0.98$	$p = 0.0034$<br>$d = 1.09$	$p = 0.0223$<br>$d = 0.74$
$dev5 > dev6$	$p = 0.0028$<br>$d = 1.80$	$p = 0.0027$<br>$d = 2.37$	$p = 0.0027$<br>$d = 2.12$	$p = 0.0035$<br>$d = 2.21$	$p = 0.0032$<br>$d = 1.56$
$dev6 \neq std2$	$p = 0.6009$<br>$d = 0.11$	$p = 0.7961$<br>$d = 0.23$	$p = 0.6820$<br>$d = 0.37$	$p = 0.0395$<br>$d = 0.69$	$p = 0.4662$<br>$d = 0.48$

Table S1: **Statistics of the group-level Wilcoxon sign rank tests for the pure tone data.** The indicated hypotheses refer to the alternative hypotheses of the tests. The comparison of $dev6$ and $std2$ was conducted using two-tailed sign rank tests; all other contrasts were tested using one-sided sign rank tests. All p-values were corrected for 30 comparisons using the Holm-Bonferroni method. Effect size d: Cohen's d.

Hypothesis	Te1.0 L	Te1.0 R	Te1.1 L	Te1.1 R
$std0 > std2$	$p = 0.0057$<br>$d = 3.33$	$p = 0.0057$<br>$d = 2.95$	$p = 0.0051$<br>$d = 4.41$	$p = 0.0050$<br>$d = 4.07$
$dev4 > std2$	$p = 0.0077$<br>$d = 2.66$	$p = 0.0104$<br>$d = 2.45$	$p = 0.0055$<br>$d = 3.44$	$p = 0.0049$<br>$d = 3.24$
$dev4 > dev6$	$p = 0.0060$<br>$d = 2.91$	$p = 0.0114$<br>$d = 2.26$	$p = 0.0056$<br>$d = 3.46$	$p = 0.0054$<br>$d = 3.29$
$dev4 > dev5$	$p = 0.0074$<br>$d = 1.08$	$p = 0.0100$<br>$d = 0.94$	$p = 0.0053$<br>$d = 1.26$	$p = 0.0059$<br>$d = 1.35$
$dev5 > dev6$	$p = 0.0056$<br>$d = 2.51$	$p = 0.0109$<br>$d = 1.91$	$p = 0.0052$<br>$d = 3.02$	$p = 0.0051$<br>$d = 2.84$
$dev6 \neq std2$	$p = 0.3888$<br>$d = -0.48$	$p = 0.3720$<br>$d = -0.25$	$p = 0.6543$<br>$d = -0.26$	$p = 0.1057$<br>$d = -0.63$

Hypothesis	Te1.2 L	Te1.2 R	Te3 L	Te3 R
$std0 > std2$	$p = 0.0107$<br>$d = 2.12$	$p = 0.0050$<br>$d = 2.35$	$p = 0.0053$<br>$d = 2.76$	$p = 0.0067$<br>$d = 2.65$
$dev4 > std2$	$p = 0.0169$<br>$d = 2.07$	$p = 0.0103$<br>$d = 1.86$	$p = 0.0096$<br>$d = 2.44$	$p = 0.0092$<br>$d = 2.52$
$dev4 > dev6$	$p = 0.0095$<br>$d = 2.25$	$p = 0.0509$<br>$d = 1.43$	$p = 0.0199$<br>$d = 1.93$	$p = 0.0159$<br>$d = 1.94$
$dev4 > dev5$	$p = 0.0104$<br>$d = 0.83$	$p = 0.0663$<br>$d = 0.48$	$p = 0.0292$<br>$d = 0.66$	$p = 0.0149$<br>$d = 0.68$
$dev5 > dev6$	$p = 0.0084$<br>$d = 2.01$	$p = 0.0319$<br>$d = 1.28$	$p = 0.0133$<br>$d = 1.90$	$p = 0.0161$<br>$d = 1.76$
$dev6 \neq std2$	$p = 0.3416$<br>$d = -0.34$	$p = 0.9183$<br>$d = 0.28$	$p = 0.0859$<br>$d = 0.51$	$p = 0.0765$<br>$d = 0.65$

Table S2: **Statistics of the group-level Wilcoxon sign rank tests for the FM-sweep data.** The indicated hypotheses refer to the alternative hypotheses of the tests. The comparison of $dev6$ and $std2$ was conducted using two-tailed sign rank tests; all other contrasts were tested using one-sided sign rank tests. All p-values were corrected for 48 comparisons using the Holm-Bonferroni method. Effect size d: Cohen's d.
